# Computer-Guided Bone Biopsy: A Technical Note with the Description of a Clinical Case

**DOI:** 10.3390/bioengineering8120214

**Published:** 2021-12-15

**Authors:** Federica Altieri, Giovanna Iezzi, Valeria Luzzi, Gianni Di Giorgio, Antonella Polimeni, Michele Cassetta

**Affiliations:** 1 Department of Oral and Maxillofacial Sciences, School of Dentistry, “Sapienza” University of Rome, 00100 Rome, Italy; valeria.luzzi@uniroma1.it (V.L.); gianni.digiorgio@uniroma1.it (G.D.G.); antonella.polimeni@uniroma1.it (A.P.); michele.cassetta@uniroma1.it (M.C.); 2 Department of Medical, Oral, Biotechnological Sciences, University “G. d’Annunzio” of Chieti-Pescara, 66100 Chieti, Italy; giovanna.iezzi@unich.it

**Keywords:** biopsy, computer-assisted surgery, CAD-CAM, CBCT, oral surgery, digital planning

## Abstract

Aim: The aim of this technical note is to present a computer-aided design–computer-aided manufacturing (CAD–CAM) surgical guide to perform a computer-guided bone biopsy. Traditionally, to diagnose abnormal conditions affecting jawbone, a bone biopsy is performed with the use of a trephine bur. The positioning of the bur, during the biopsy, is based on the skill of the surgeon; therefore, an inaccurate placement of a trephine bur may occur. The use of a guide, however, can minimize this risk and achieve a better result. Materials and Methods: To determine the site and the extension of bone sampling, the stereolithography file (STL) file of cone–beam computed tomography (CBCT) images is acquired using a specific planning software and superimposed with the STL file of a dental cast; a virtual surgical guide is designed, using the same software that allows a 3D (three-dimensional) view of the guide from different perspectives and planes. The number and site of guide tubes are determined on the basis of the width and the extension of the sampling; thanks to a 3D printer, the surgical guide is manufactured. Results: The use of a customized surgical guide realized with CAD–CAM technology allows a precise and minimally invasive approach, with an accurate three-dimensional localization of the biopsy site. Conclusions: The high precision, great predictability, time-effectiveness and versatility of the present guide should encourage the clinician to use this minimally invasive surgical approach, but controlled clinical trials should be conducted to evaluate the advantages as well as any possible complications.

## 1. Introduction

An accurate diagnosis and treatment of oral disease is an essential component of the patient’s comprehensive dental care and the foundation of high-quality dentistry. Few bony abnormalities can be accurately diagnosed based on their radiographic features [1]. Confirmatory diagnosis may require a biopsy and microscopic examination [2]. In current dental practice, the reconstruction of cranial and maxillofacial defects is made through the use of bone grafting. This is a surgical procedure that replaces missing bone with autologous bone, or with an artificial, synthetic, or natural substitute [3]. The most common use of bone grafting is in implant-supported rehabilitation, in order to restore the edentulous area of a missing tooth [3]. Once the transplanted bone is secured into its new location, the blood supply is generally restored to the bone on which it is attached. To evaluate the graft histology characteristics a bone biopsy is necessary [2]. Today, the most precise imaging to perform a thorough evaluation of the maxillary bone structure is cone-beam computed tomography (CBCT), which provides high spatial resolution, accessibility and a lower radiation dosage compared to computed tomography (CT) [4,5]. 

The aim of the present technical note is to describe a computer-aided design–computer-aided manufacturing (CAD-CAM) surgical-guide procedure used to perform a bone biopsy after a previous bone graft; a single case of guided bone biopsy with subsequent implant insertion is described. 

## 2. Materials and Methods

The present procedure, used at the Department of Oral and Maxillo-Facial Sciences of ‘‘Sapienza’’ University of Rome, allows jawbone biopsy employing a surgical guide realized with at least one tube that guides a trephine bur to enable accurate bone sampling of the maxillary and/or mandibular bone. The realization of the present surgical template requires:

- The determining of bone sampling site and extension. For this purpose, DICOM (Digital Imaging and Communications in Medicine) files of CBCT are acquired using specific planning software and a superimposition of a 3D (three-dimensional) scan of the cast model (Easy Optical3D Scanner, Open Technologies, Rezzato, BS, Italy) is performed after uploading the corresponding STL file (stereolithography file);
- The design of a virtual surgical guide through CAD software that allows a view of the 3D guide from different perspectives and planes; the number and site of guide tubes are determined on the basis of the width and the extension of the sampling;
- The surgical guide printing, thanks to a 3D printer (Stratasys OrhoDesktop, Eden Praire, MN, USA).

## 3. Description of a Computer-Guided Biopsy 

### 3.1. Manufacturing the Surgical Guide

In a young adult patient with the need for prosthetic rehabilitation of the maxillary arch, due to multiple dental agenesis, the initial phase of treatment involved a split crest procedure using autologous bone grafting. In order to achieve an adequate implant, osseointegration and a successful treatment outcome from both functional and aesthetic points of view are required, as is a good dental emergence profile. Equally, to obtain a correct prosthetic rehabilitation [6] it is important to have at least 2 mm of width around the implant bone crest at the buccal and palatal planes. Intra-oral tissues (mandibular branch) or the extra-oral tissues (e.g., iliac crest bone) grafts usually led to good results, but they are invasive and complications cannot be excluded, such as additional surgical procedures [6]. To find an alternative solution in such cases, techniques for crest expansion using bone expanders or osteotomes, or “split-crest” (SCT) performed with an ultrasound device or with conventional surgery have been proposed [7]. The “split-crest” technique consists of splitting the vestibular and oral cortical bones, displacing the vestibular cortical bone in either the maxillary or mandible bone and separating it from the bone marrow and creating a middle gap, which is usually occupied mostly by the inserted implants. The space unoccupied by the implants can be filled with biomaterials such as autologous bone grafts, particulate bone, or plasma derivatives, such as platelet-rich plasma [7]. 

**Figure 1:**
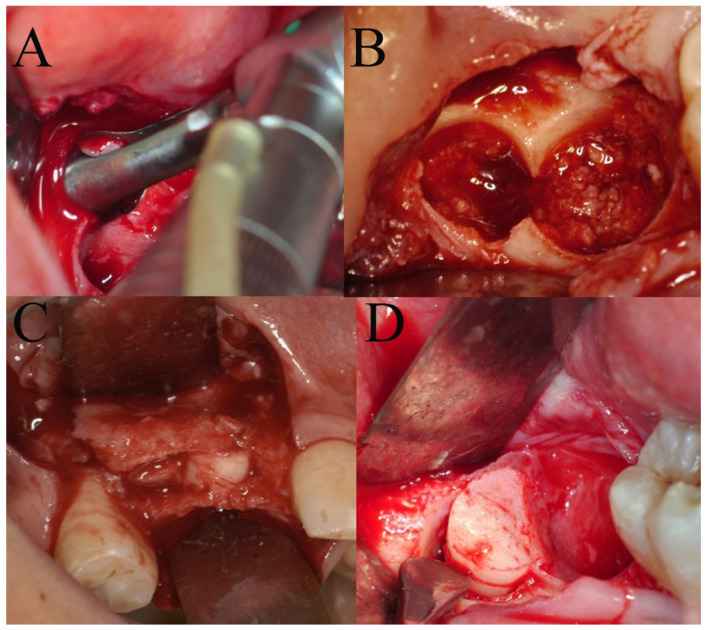
(**A**) Bone harvest from the retromolar trigone with the use of a trephine (4 × 6 mm) bur; (**B**) a detail of the bone sampling site; (**C**) the bone defect obtained by the separation of the bone segments is filled with bone chips, which were condensed in the space between the buccal and palatal bone plates with the aim of completely filling the space; (**D**) a detail of the bone graft.

**Figure 2:**
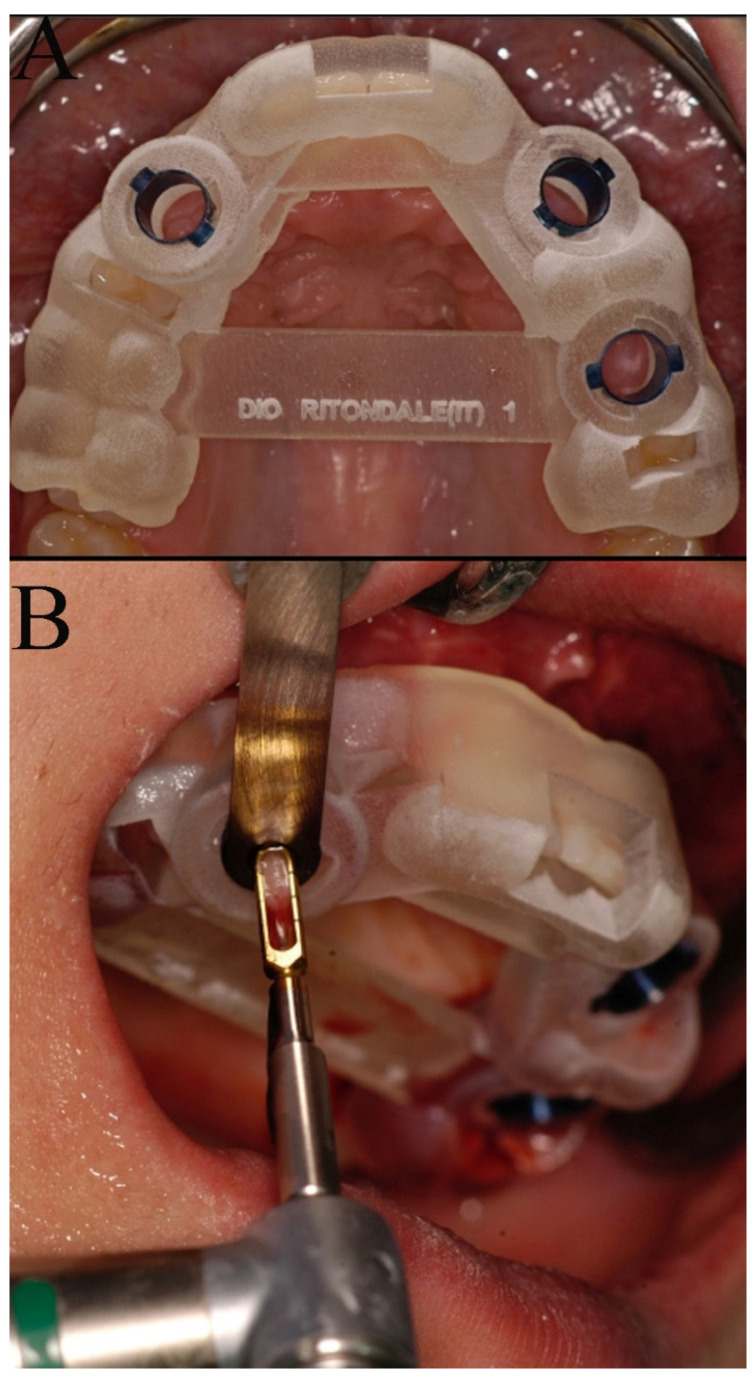
(**A**) The tooth-supported surgical guide positioned in the upper arch; (**B**) a detail of the biopsy trephine bur.

**Figure 3:**
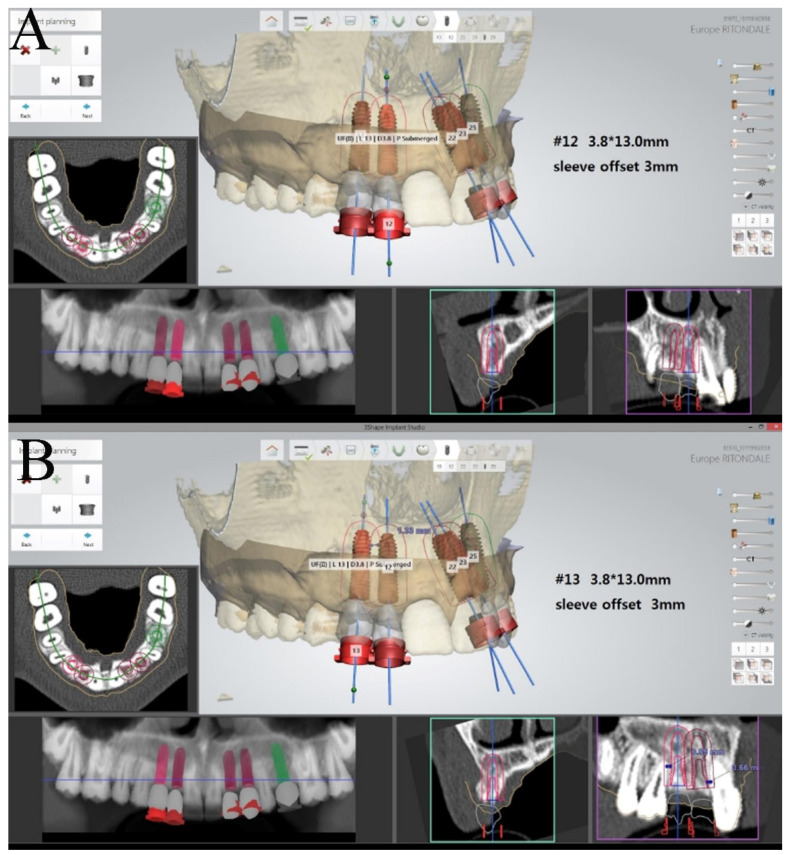
(**A**) Virtual implant planning of 1.2 on the 3-Shape Implant Studio software; (**B**) planning of implant position in the canine area [1.3].

The surgical sites were assessed by a clinical intraoral examination, panoramic and periapical radiographs. The split crest criteria were used for the patient’s evaluation: (1) a minimal horizontal bone width of 2 mm; (2) a minimal vertical bone height of 10 mm; (3) no concavity in alveolar bone profile; and (4) the horizontal osteotomies had to end at least 1 mm distance from the neighboring teeth [8]. A CT was requested for a 3D pre-operative evaluation to determine the presence of an alveolar width of at least 2 mm and the absence of a concavity. A staged rehabilitation approach was planned given that the two-stage ridge split usually has a higher implant success rate when the ridge width is lower than 5 mm after splitting [9]. After local anesthesia (Optocain^®^-Molteni Dental-Italia), a full thickness crestal incision extended buccally and palatally was made with vertical divergent releasing incisions extended into the vestibule. A mucoperiosteal flap was elevated, and the bone ridge was exposed. The cortical bone was initially curetted, to remove all residual connective tissue and periosteum, then, using a piezoelectric scalpel, a horizontal incision was made in the middle of the ridge with two releasing incisions, one mesial and one distal. The horizontal osteotomies were performed at a distance of at least 1 mm from the neighboring teeth. The alveolar ridge was split longitudinally in two parts, provoking a greenstick fracture using a 4 mm straight osteotome (Hu-Friedy Mfg. Co., Chicago, IL, USA). The straight osteotome was gently tapped on with a hammer to create a fine cut longitudinal to the crest. The osteotome was then used as a lever to spread apart the two cortical plates. The surgical fracture was extended to a depth of 10 mm. Many attempts were made to avoid sharp and complete vertical or horizontal fractures of the buccal and palatal bone plates. After a crestal incision, bone from the retromolar trigone was harvested using a trephine (4 × 6 mm) and then fragmented into particles (bone chips). The bone defect obtained by the separation of the bone segments was filled with bone chips, which were condensed in the space between the buccal and palatal bone plates with the aim of completely filling the space (Figure 1). The mucoperiosteal flap was sutured using tension-free single sutures (GORE–TEX, W.L.Gore and Associates, Inc., Flagstaff, AZ, USA). Suture removal was performed 10 days after the surgical procedure. A reduced implant placement time approach in the staged rehabilitation was used [10]. After a healing period of 2 months, a computer-guided implant surgery was carried out. During the computer-guided procedure a bone graft sample was harvested thanks to a computer-guided biopsy (Figure 2). A dedicated CAD software ( 3Shape Implant Studio^®^, Srl Milam, Italy ) was used to plan the number, the length and the diameter of dental implants as well as the dimensions of trephine bur to be used (Figure 3). A software application allowed the design of the surgical guide (3Shape Implant Studio^®^) printed with a 3D printer (3D Stratasys OrthoDesktop) (Figure 2A). In the present case two surgical guides were realized with a total of 5 tubes. Since the diameter of the tubes is larger than that of the drills and the implant, in some cases it is necessary to utilize two guides to hold two contiguous pipes. 

It was not possible to design a single surgical guide due to the limited space in the dental arch and the size of the tubes. The tubes guided the trephine bur as well as the implants into their planned positions. Using dedicated sleeves of different diameters, it was possible to guide, first, the trephine bur and then the implant drills and the implant mounting devices. Bone cores were harvested using a 3.5 × 10 mm diameter trephine bur under saline solution irrigation and processed for histology. Every guide had different tubes called “master tubes” embedded within the acrylic resin surgical guide; the master tubes had cylindrical walls and wings to prevent their rotation and to provide greater mechanical strength. To adapt the master tube to the trephine bur, to the implant drills and also to the mounting devices, removable sleeves were used. The removable sleeves have a variable inner diameter that permits the accommodation of the biopsy trephine bur, the implant drills, and the implant mounting devices.

The tooth-supported surgical guide was constructed with a 3D printer (3D Stratasys OrthoDesktop).

### 3.2. Specimen Processing 

A total of four bone cores, corresponding to the four implant sites planned in the grafted bone tissue, were retrieved and immediately stored in 10% buffered formalin. The specimens were processed using the Precise 1 Automated System (Assing, Rome, Italy). The specimens were dehydrated in a graded series of ethanol rinses and embedded in a glycolmethacrylate resin (Technovit, Kulzer, Wehrheim, Germany). After polymerization, the specimens were sectioned, along their longitudinal axis, with a high-precision diamond disk at about 150 lm and ground down to about 30 lm with a specially-designed grinding machine. Two slides were obtained from each specimen. These slides were stained with acid fuchsin and toluidine blue and examined with transmitted light Leitz Laborlux microscope (Leitz, Wetzlar, Germany). Histomorphometry of the percentages of newly formed bone, residual grafted material, and marrow spaces was carried out using a light microscope (Leitz) connected to a high-resolution video camera (3CCD, JVC KY-F55B, JVC, Yokohama, Japan) which interfaced with a monitor and PC (Intel Pentium III 1200 MMX, Intel, Santa Clara, CA, USA). This optical system was associated with a digitizing pad (Matrix Vision GmbH, Oppenweiler, Germany) and a histometry software package with image capturing capabilities (Image-Pro Plus 4.5; Media Cybernetics Inc., Immagini & Computer SncMilano, Italy).

### 3.3. Histological Results

**Figure 4:**
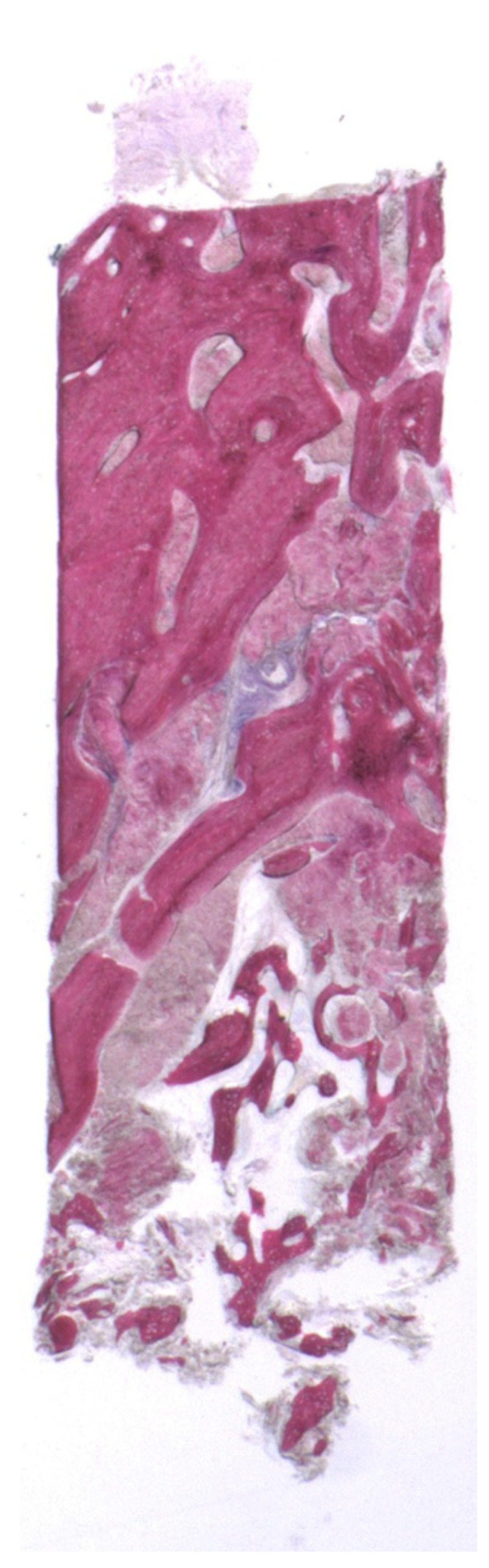
At low magnification, two areas with different features are observed: the coronal one, characterized by trabecular bone with small marrow spaces; and the apical, where autologous bone particles surrounded by new bone can be seen (toluidine blue and acid fuchsin 9×).

**Figure 5:**
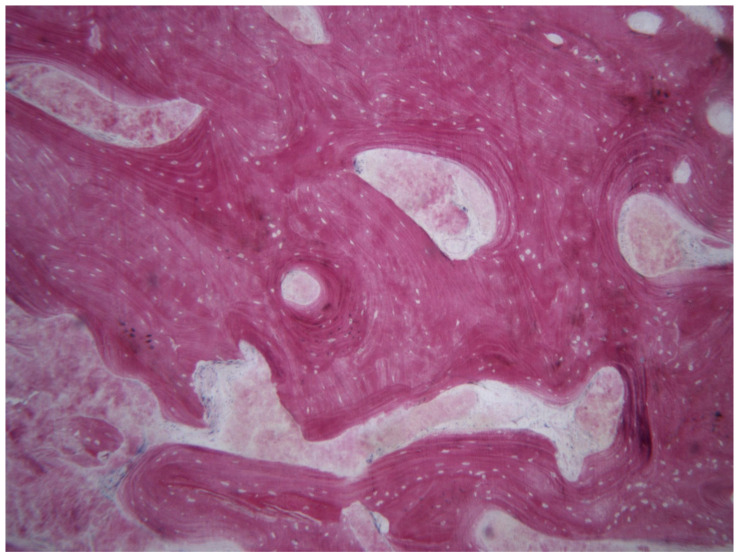
Trabecular bone with small marrow spaces. In some areas bone particles undergoing remodeling process were detected (toluidine blue and acid fuchsin 40×).

**Figure 6:**
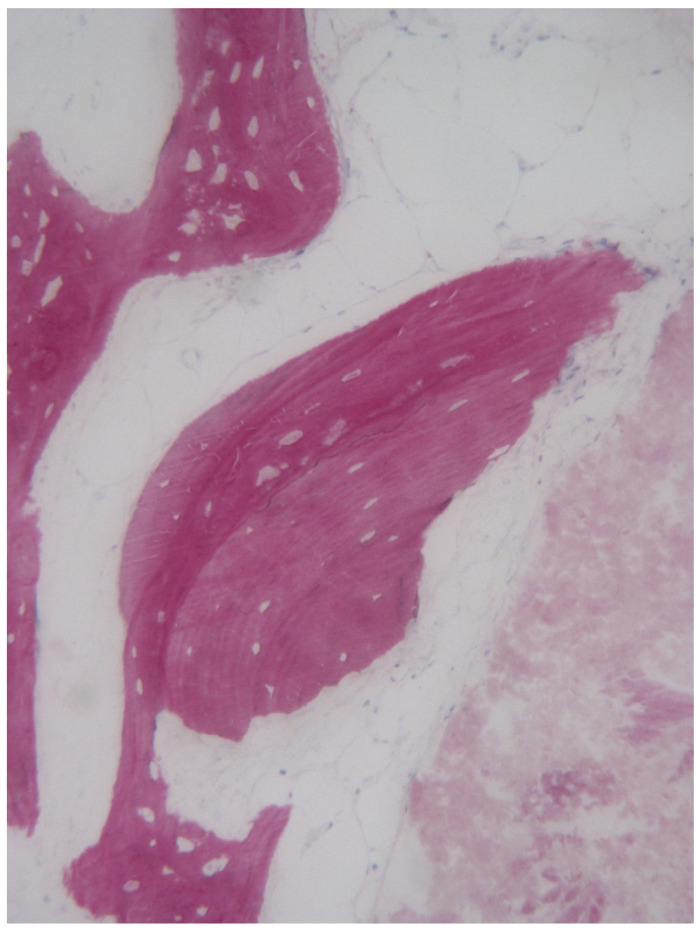
In the apical portion several residual biomaterial particles lined by new bone are found. In the marrow spaces there are many blood vessels close to the newly formed bone and the biomaterial particles (toluidine blue and acid fuchsin 100×).

At low magnification, trabecular bone with small and large marrow spaces was observed (Figure 4). The coronal portion of the alveolar bone, after split crest, was evident. In this portion the preexisting trabecular bone with small marrow spaces was in contact to many remodeling and new bone formation areas. Moreover, some residual autologous bone particles, which probably underwent a process of remodeling ending up in their replacement with new bone, could be detected (Figure 5). Instead, the apical portion of the bone core was characterized by newly trabecular bone and residual grafted particles, lined by newly formed bone, which showed the features of a recently formed tissue, such as wide osteocyte lacunae, high staining affinity, the presence of osteoblasts and the osteoid matrix’s undergoing mineralization. In many areas the collagen matrix undergoing a remodeling process was present. In the marrow spaces there were some blood vessels close to the newly formed bone and the biomaterial particles (Figure 6). Inflammation and multinucleated giant cells were absent. Histomorphometrical analysis showed that the percentage of newly formed bone was 22.8%, marrow spaces 60.1% and residual grafted material 17.1%. 

## 4. Discussion

Given the differences in treatment and prognosis for many bony entities, the identification of these lesions mandates biopsy. When radiological findings in the maxillary and mandible bones require a histopathological examination for the correct diagnosis, computer-guided biopsy can be used. Further treatment, if necessary, will then be dictated by the definitive histopathologic diagnosis. The guide described in this note can be used for many purposes: not only to guide the bone biopsy but also the implant site preparation and the implant insertion [11]. This new technique allows both bone harvesting and implant insertion to be performed at the same time. Bone sampling and subsequent histological analysis allow us to evaluate the results obtained following bone regeneration. This technique can also be extremely useful in case of research aimed at comparing the images of a CT or CBCT with the corresponding anatomical structure, allowing the clinician to obtain a match between the radiological and the histological images. This technique can be used even if no implants have to be inserted, i.e., it can be used to perform solely the biopsy. The surgical guide can be tooth-, bone- or mucosa-supported.

The present computer-guided biopsy seems to have many advantages: a precise and reliable biopsy sampling, a reduced surgical time, and safety of the anatomical structures. As this procedure is flapless and minimally invasive, it reduces the post-operative discomfort; in addition, it is a safe method to prevent neurological damage in complex anatomical regions with proximity to nerve branches. On the other hand, this procedure requires the use of dedicated software and the construction of a surgical guide is expensive.

Moreover, considering the results of a recent study aimed to determine the presence of a learning curve in static computer-assisted surgery (s-CAS), it would seem possible to obtain predictable results in terms of accuracy from the beginning, using a CAD–CAM surgical template. In fact, s-CAS it is not characterized by a typical “learning curve” [12].

## 5. Conclusions

In conclusion, the high precision, great predictability, time-effectiveness and versatility of the present guide should encourage the clinical use of this minimally invasive surgical approach, but controlled clinical trials should be conducted to evaluate the advantages of the current method concerning treatment time and patient discomfort, as well as possible complications.

## Data Availability

The study did not report any data.
